# The Transactions of the Provincial Medical and Surgical Association. Vol. XIII

**Published:** 1845-10

**Authors:** 


### THE TRANSACTIONS OF THE PROVINCIAL MEDICAL AND SURGICAL ASSOCIATION. Vol. XIII. 1845. 8vo. pp. 417.

THE present volume of these "Transactions" is not equal, in the interest and importance of its contents, to some of its predecessors, although much superior to them in all that relates to the "getting up." There are five communications.—1. A Retrospective Address, by Dr. Cowan, upon the Progress of Practical Medicine. 2. A Retrospect of Anatomy and Physiology for 1843-4, by Dr. Budd, of Bristol. 3. A Paper upon Inflammation of the Retina, by Dr. Hocken. 4. An Essay upon Inversion of the Uterus, by Mr. Crosse, of Norwich; and 5. A Case of Malformation of the Urinary Organs, by Mr. Giles of Stourbridge.

The two Retrospects are ably written, especially that by Dr. Budd; but treating of by-gone things, most of which have already come under our review, they demand no detailed notice at our hands. There are, however, some of the introductory and incidental observations which we are desirous of placing before our readers.

The following passages exhibit Dr. Cowan's view of the present position of medical science.

"The science of medicine may be said to be now emerging out of infancy into manhood, to be gathering up the copious generalisations of the past, and submitting them to the ordeal of inductive examination, to be testing hypothetical assertion in the stern crucible of experiment and observation, and to be giving to language an accuracy and a limitation of which before it was insusceptible. We are, in fact, engaged in reducing an art into a science, and are arrived at the period of maximum difficulty and toil. The elements in our hands are fashioning and shaping for future forms; but, though we can vision the dim outline of a higher and nobler erection, there is yet too much movement and bustle for accurate measurement of what we are accomplishing; things new and old are strangely commingled, and impediments arise from the very number and zeal of the artificers. There never was a period of such sudden excitement, such extensive and simultaneous activity. The unexampled development of the contributory sciences has furnished fresh and unexpected instrumentality for pathological inquiry, and compelled renewed investigation in accordance with the nature of the means now in our possession."

After alluding to the effect which recent chemical and microscopic research, and the revived attention to humoral physiology and pathology have had in indicating the defects of our already acquired information, and in at once extending our sphere of acquirement and the number of our resources, Dr. Cowan continues:—

"Another most striking feature of our present condition is the unexampled facility afforded by the press for the expression and diffusion of thought. Every idea now finds an easy channel for utterance; in our periodicals and other publications encouragement is given for the most fleeting expression of opinion; and investigations, whether trivial or important, are equally honoured by publicity. The necessary consequence has been, that evil has been mingled with the good; our literature has become most inconvenient from its bulk, full of crudities and endless repetitions, heterogeneous and fragmentary in character. The student is wearied and confused by the very extent of the surface, the exercise of thought is impeded by so continued a draught upon his powers of acquisition, and what is original and vigorous in the mind is too often repressed or entombed. The natural tendency upon an ambitious spirit, if not of the highest and most comprehensive order, is to over-stimulate and exhaust its energies in the mere effort to acquire, to consume beyond the power of digestion, and either to rest with slavish dependence upon the dogmas of others, or escaping from what is felt impossible to reconcile, to pursue the path of ignorant self-reliance, reckless of all authority and experience.

"We are kept in a constant state of expectancy and interrogation: doubt pervades every thing, and truths which ages have confirmed are exposed to assailants of every form and dimension, boldly requestioned and challenged by the experimenter of a day. Our periodical literature, and more formal treatises, inconveniently and undesirably multiplied, teem with detail and assertions of the most superficial and contradictory character; yet all, as in olden times, based upon *facts,* and raised to a higher nominal authority, because supposed to be founded upon a purer indicative philosophy."

That the great facilities, and even inducements, which the numberless medical periodicals now offer for whosoever is willing to appear in their columns (whether in the shape of an original article, a case with endless observations, observations without any case, or in the hundred ways in which their voluminous pages are now filled), are attended with considerable evils, there can be, we suppose, no doubt. In this way, the superficial and incompetent are placed in an unduly prominent position, a feverish anxiety for premature publicity is engendered, and even the really competent are deterred from bestowing that thought, care, and re-examination of their productions, which would be considered but due were they destined to appear in a less fleeting medium. But, on the other hand, we must not forget that, without such facilities, many really valuable communications would have never seen the light, and that they form an excellent means for the wide diffusion of the knowledge of the improvements in the more practical portions of our art. We may observe, *en passant*, that the truth enounced by the Reporter in a subsequent part of his address—" A much smaller number of medical periodicals, if truthfully and energetically conducted, might be most advantageously substituted for their, at present, most inconvenient multiplication"—does not appear with the best grace when addressed to the proprietors of one of the youngest of the family—whose pages, too, have been subsequently opened for the reception of articles in defence of the ultra-pretensions of Mesmerism.

Notwithstanding the, in some respects, chaotic state of our accumulating materials, Dr. Cowan justly observes, that our condition upon the whole is one of progress.

" While making these remarks, we are not overlooking the far more gratifying and unquestionable consideration, that medicine, both as a science and an art, is really advancing; that the labours of a few master-spirits, particularly during the past half-century, have astonishingly extended our knowledge of the nature and seat of disease; that we are in the possession of a discriminating power in diagnosis and treatment, to which our predecessors were strangers; and that, provided as we are with new instruments of research, and furnished with additional therapeutic resources, we may yet calculate upon much increase beyond what has already been attained. But, still, it is no less true that we have our disadvantages, and there are clouds which bedim the horizon of our future prospects.

" We are too apt to forget that the great work we are now engaged in is rather that of explanation or development, than of discovery; the clearer illustration of what is old more than the revealing of what is new; reducing into accuracy of classification and expression what was before only general and vague, and demonstrating the soundness, not the absurdity, of many of the ideas and conclusions of our predecessors. We must not estimate our present progress by mere numbers, opportunity, or zeal, nor suppose that the multitude will ever be directly instrumental in the extrication of truth. No: this holy and glorious mission will ever be the privilege of the gifted and laborious few, and its accomplishment be impeded rather than advanced by the undue multiplication of labourers. However excellent the system we adopt, however perfect the scheme of our enquiries, the true value of results must ever chiefly depend upon the power and qualifications of the observer. Few, very few, possess these to the requisite extent, and hence we are daily overwhelmed with materials too incomplete and amorphous ever to become the foundation of scientific induction."

There are some useful observations also upon the inefficiency of the *numerical method* as applied to practical medicine. The ever-changing and complex character of the phenomena of disease render resort to it impossible or unsafe.

" Though strongly advocating the utmost possible extension of numerical research, substituting as it does the accurate for what is vague, and regarding its varied and extensive applications at the present moment as among the distinctive peculiarities of our times, we yet feel that in the practice of medicine its uses are limited, and that there are evils as well as benefits resulting from its undue application. Not only is it incapable of preserving us from mistakes as egregious as any which have been fostered under the less definite sanction of experience, but it imparts to falsehood a fearful semblance to truth, and gives to assertion an absoluteness and precision well calculated to satisfy the inquirer, and repress the effort of independent observation."

Dr. Cowan administers a deserved reproof to those who, dazzled by our apparent progress, perceive not any obstacle to its continuance, and are prone to contrast it with by-gone times. He maintains we have not possessed ourselves "of a single leading principle not recognized by the soundest thinkers of ages which are past," while we have not escaped "a warfare of hypotheses as violent and contradictory as any which has characterized the darkest periods of our history."

We will now make a few extracts from the body of the address.

*Purgatives in Fever.*—" The employment of purgatives in Fever has met with an intelligent investigator in Mr. Hunt. He very rightly considers many of the functional disturbances to have a remedial tendency, and objects to the routine interference with a torpid state of the bowels in the low stages of fever, regarding the condition as favorable to digestive and nutritive repair. He has known seven days to elapse without an alvine evacuation, and with the greatest apparent advantage, and we believe many practitioners have had to regret the exhibition of even the mildest laxative in the later stages of fever, accompanied with much abdominal irritation. Endeavouring, by alterative and laxative medicine, to render the excretions healthy, must necessarily be fruitless, if not injurious, so long as the entire system is specifically disturbed, and yet it is often attempted, as if the individual were otherwise healthy. To proscribe laxatives altogether, would be equally undesirable. There are many degrees between the systems of Hamilton and Broussais, but in England we are perhaps too much impressed with the necessity of regular alvine discharges under all circumstances, and sometimes forgetful of the peculiarities of diseased existence. The right use of purgatives in fever is unquestionably a point of great practical difficulty."

*Follicular Ulceration in Fever.*—" We have never been able to partake of the enthusiasm excited by the discovery of intestinal follicular changes, and have often felt wearied at their minute and endless description, unable to grasp the immense importance of the fact, or to perceive its asserted bearings upon the explanation or pathology of fever. That it is desirable to know every change which disease induces in our organs, no one can reasonably deny, and in proportion as pathology is accurate is our treatment successful; but the tendency to over-estimate them has been strikingly displayed in modern times, and incalculable labour has been devoted to subjects of very secondary importance. Such views may be regarded as heretical by many, but of their ultimate prevalence and truth we feel confident.

*Buffy Blood.*—" Mr. Wharton Jones has pointed out a very ingenious method of determining whether the blood is buffy or not, from the examination of a very minute portion of this fluid. It consists of quickly enclosing a drop between two pieces of glass, and observing with the naked eye the quickness with which it assumes a mottled appearance, and the smallness or largeness of the interspaces. In buffy blood the mottling is almost instantaneous, and the interspaces large, while in healthy blood it is delayed for half-a-minute or more, and the reticulation is minute."

*Nature of Insanity.*—The following are some of the remarks made by Dr. Cowan, when referring to the Rev. J. Barlow's essay, "On Man's Power over Himself to prevent or control Insanity."

" The tendency of recent research has certainly been to exalt the material at the expense of what is spiritual: to make much of the instrument, and but little of the controlling power; to regard, in short, mental aberrations too exclusively as the expression of organic disturbance. Much practical good has undoubtedly resulted from the clearer apprehension of the truth, that all mental and moral manifestations are effected through the medium of a special organization, and therefore dependent for their exercise on the health and integrity of the instrument; but, while fully admitting such a dependence, and giving due credit to those who have laboured most successfully for its establishment, we have felt conscious that the agency of mind upon matter was virtually, though not always nominally, disregarded; and that the actual increase to our knowledge resulting from cranioscopical examination, was really much less than what was confidently assumed. * * * * * * *
No man can doubt, if deducing his conclusion from the knowledge of his own heart, that his mental aberrations from what is right, are moral not cerebral delinquencies; and if so, why should we shrink from admitting the possibility of morbid manifestations sometimes originating in a perverted spiritual principle, as well as in diseased organization? Such a supposition is consistent with the statements and facts of revelation; with what we know of the daily and hourly operations of our own mind; and will, we feel confident, be increasingly acknowledged in proportion as we ascend to a sounder and more comprehensive philosophy."

*Epilepsy.*—" Dr. Wakefield Scott has adduced some additional cases confirmative of the value of large doses of *Digitalis*, as advised by Dr. Starkey and others; and M. Semoine states that he has successfully treated three confirmed cases by the following mixture, the value of which can only be inferred from the assertion of its efficiency. ℞. Liq. Amm. Ac. ℥xij. Syr. Aur. ℥j. Aquæ ℥ij. Aq. Lauri-ceras. ℥j. S. coch. amp. iij. in die. Nothing can be more vague and unsatisfactory than the generality of reported cures of epilepsy. Very careful discrimination, and very numerous and long-continued experiments would be necessary to arrive at any trust-worthy results. The clearer appreciation of reflected sources of cerebral irritation appears to be the principal advance which modern investigation has made in the diagnosis and management of this most distressing complaint."

*Naphtha in Phthisis.*—" After the statements of Dr. John Hastings, the long-sought remedy might have been considered as at last discovered. But, alas! naphtha, like its shadowy predecessors, seems destined to equal, if not to a more speedy, oblivion. Two or three faint testimonies in its favour have been recorded during the past year, but the evidence of its entire unworthiness to merit the praises so injudiciously bestowed, is too general and decided to admit of reasonable doubt. Naphtha may, and probably has its uses, but as a *cure* for consumption, it is utterly to be discarded. We have ourselves rather extensively tried it, and have never derived a single advantage from its use."

*Ovariotomy.*—Although we have stated our arrival at a different conclusion in former numbers of this Journal, we are glad to be put into possession of the opinion of so able and dispassionate an observer as Dr. Cowan upon this much-controverted operation.

" From the admirably detailed cases of Dr. Clay, Dr. Walne, Dr. F. Bird, and Mr. Southam, besides other instances reported from the Continent and America, not to mention the statements made by earlier observers (for the operation dates back at least 70 years), we believe the value and rationality of extraction, under favourable circumstances, to be satisfactorily demonstrated; and in the statistics of the operation, so diligently collected by Mr. Philipps, Dr. Churchill, and a writer in the British and Foreign Medical Review, we can discover nothing that would justify a more unfavourable conclusion, because materials of a most dissimilar and heterogeneous character form the elements of their calculations, and are utterly incapable of eliciting truth. The numerical system so applied becomes a positive abuse, and gives a sanction to results which have nothing to recommend them but the garb of arithmetical expression. If the attendant risk, when properly performed, be not greater than that of major operations generally, and if the inefficiency of internal treatment be ascertained, we regard all discussions on diagnosis and methods of performance as of very secondary interest, because every case must, after all, be individually considered, and the same general principles must be acted upon as would influence our decision under other, but analogous, circumstances."

The misfortune is, that, in the greater number of instances, the extirpation has been undertaken in complete violation of the "same general principles" which are our guide in determining upon the performance of other capital operations; and the consequence is, that in the history of no other operation will life have been found so frequently jeopardized and lost for the removal of a disease not necessarily, or at all events speedily, mortal; or the surgeon so frequently discomfited by discovering, when in the middle of his painful and formidable undertaking, that the disease for which it has been put into force exists not, or is of such a nature as to render the completion impossible.

*Quackery.*—Dr. Cowan has long fought a good fight against empiricism in its various forms, and we regret to find he almost thinks the cause is a desperate one.

"We much regret that, in the measures contemplated by Government, there is no indication of any intention actively to legislate against the unqualified practitioner, or to suppress or regulate the sale of empirical nostrums. We confess ourselves weary with the effort to rouse a feeling of bold resistance to quackery in every form, and to popularize the conviction that it ought to be restrained by the strong arm of the law. On what principle law is to prove inoperative for such a purpose we have never been able to comprehend: and to expect the remedy in a diffusion of knowledge, or in the good sense of those who never can and never will appreciate the true merits of the question, we can only regard as a vain and hopeless chimera."

Dr. Cowan suggests, among other means of discountenancing quackery, "that the press should not be a sanctioned vehicle for announcements which are palpably false and necessarily injurious; that men who are manifestly disregarding the first principles of truth, and making merchandise of public ignorance and credulity, should not be assisted in the diffusion of falsehood by agents intended only to benefit and enlighten." The advertisements of quacks and quack medicines form by far too profitable a source of emolument ever to be relinquished by the proprietors of newspapers, especially the lower class of these. Their forcible suppression is obviously impossible, and the imposition of heavy duties upon this class of announcements would be liable to the very "Government participation" which is objected to, by the author and others, in regard to the medicine stamp-duty. In France, where the laws governing the press are sufficiently arbitrary, those against quackery much more stringent, and the anxiety of the State much more unequivocal than with us, the advertising columns of the newspapers teem with announcements as delusive and preposterous as do our own. The noble stand recently made by the "Nation" newspaper in Ireland, seems to have been but coldly responded to by the profession.

Dr. Budd's *Retrospect of Anatomy and Physiology for the year* 1843-4, consists of an able critical, and in some respects analytical, review of the numerous publications upon animal chemistry and structural anatomy which have appeared during that period. Its condensed form forbids other extract than one or two collateral passages.

Of the pursuit of Physiology, independently of its connection with medicine, Dr. Budd thus expresses himself.

"The condition of physiology as a branch of knowledge—as an able writer has lately remarked of politics—was, up to a late period, that which Bacon animadverted upon as the natural state of the sciences, while their cultivation is abandoned to practitioners; not being carried on as a branch of speculative inquiry, but only with a view to the exigencies of daily practice, and the *experimenta fructifera*, therefore, being aimed at almost to the exclusion of the *lucifera*. To this circumstance, quite as much as to the inherent and peculiar difficulties attending all investigation into the phenomena of life, must be ascribed the slow progress of physiology, while it was cultivated, more or less, with a view to its subservience to the practice of medicine.

"There is, still, too general a disposition among medical practitioners to rate discoveries in this science in accordance with their immediate and obvious applications to the healing art. But surely the adoption of such a low standard of value implies great short-sightedness, and still greater ignorance of the history of science; as if the more general and abstract the truth, the greater were not, of necessity, the number of practical consequences it involves, and the greater, ultimately, the practical gain. Were it needful, a thousand instances might be brought from the annals of invention to illustrate this truth."

After observing that the adoption of this spirit of abstract inquiry in the various branches of human knowledge marks the transition from an art to a science, and has always been the herald, or rather the cause, of brilliant discoveries, Dr. Budd adds :

"In the study of the phenomena of life, the change was slow to make. Happily, however, for the advancement even of practical medicine itself, physiology is now extensively cultivated as a pure science. Numberless works of great merit are written upon it; chairs are instituted for the teaching of it; endowments are made in support of its votaries; and, in all civilised countries, numbers of eminent men dedicate their lives to its advancement. Thus, in this branch of human knowledge also, the division between the practical and the speculative, the *experimenta fructifera* and *lucifera*, is final and complete."

*Cruel Experiments on Animals.*—We cordially join with Dr. Budd in reprobating the utter recklessness with which so many of the French physiologists inflict the cruelest sufferings upon helpless animals, without the attainment, or probability of attainment, of objects of commensurate importance, or which might have been attained by less objectionable procedures. The accounts of some of these, which it has been our lot to peruse, are sickening in the extreme, from the wantonness with which they have been undertaken, and the unnecessary suffering their mode of performance gives rise to. That experiments are quite justifiable when a sufficient case for their institution is made out, we are persuaded; but, that a deep responsibility rests with individuals who undertake their performance can any one doubt. Inquirers in our own country are by no means free from the stain of having unnecessarily multiplied these severe and generally imperfect expedients; but certainly, here at least, they must yield the palm to their continental brethren. It would be bad enough were only insufficient yet accurate results derivable from these procedures; but when we find that these, as observed by different experimenters, are often of the most discordant and opposite character, only agreeing, in fact, in the amount of animal suffering they have given rise to, every heart, not already steeled by frequent perpetrations of this kind, must revolt against their continuance, except under well-defined and exceptional circumstances.

The experiments which have given rise to Dr. Budd's animadversions are those of *M. Chossat,* detailed in his work, *Recherches Experimentales sur l'Inanition,* which obtained a Monthyon prize. His plan consisted in starving a number of animals to death, and accurately recording the various phenomena as they arose.

" To us, however, it appears at first sight as nothing less than wanton cruelty of the worst kind, to starve animals to death merely to have an opportunity of observing the phenomena of inanition, when these phenomena are, unfortunately, daily exhibited to us on a large scale, in the effects of a variety of wasting diseases, and though more rarely, yet in all the simplicity of experiment, by the accidents of famine, shipwreck, and other forms of human calamity."

This, the *prima facie* view of the matter, is confirmed by the examination of the results at which this experimenter arrived—results which were already acquired by, or might have been deduced from, other more accurate and unexceptionable sources of information.

" After these remarks I need scarcely add that I am at a loss to know on what scientific ground the French Academy thought fit to confer so high and valuable a mark of distinction on this memoir. For thus giving, with so little to justify them, such an effectual encouragement to cruelty of the worst kind, they deserve the grave rebuke of all humane men. I am not one of those who entertain what may be held to be maudlin and sentimental objections to experiments on living animals; but, while freely admitting that such experiments are justifiable, where we are warranted by previous and well-founded induction in the expectation of results of importance to the well-being of man, attainable by no other means, I the more feel bound, on the part of all true lovers of science, to enter an indignant protest against such wretched cruelties as these, undertaken almost at random for the chance of what may turn up, and for the production of phenomena which accident and disease are daily offering to our observation on a large scale, and into the nature of which science has already given us the clearest insight. I want words to express my own abhorrence of these practices, and my deep sense of the guilt of those who resort to them. The frequency of them in France, and the shocking levity with which they are undertaken, is the great blot on the present character of physiological science in that country."

After reviewing the progress of organic chemistry on the Continent, Dr. Budd gives expression to the following natural regret.

" I cannot close this part of my Report without expressing my regret and humiliation at having, through its whole course, scarcely once had occasion to speak the name of an Englishman. It is true that in this province we have one or two individual names, of which we may well be proud; but we shall look in vain for anything worthy to be called a *school* of organic chemistry in this country, and still more, for anything worthy to emulate the noble schools of which Dumas and Liebig are the heads in France and Germany. I earnestly hope that we shall not long allow ourselves to be thus outstripped in this noble rivalry of nations, but that our own countrymen will strive to share in the glories that are yet in store for men of genius, in the bright career which these illustrious chemists have opened to us."

Dr. Budd truly adds that, the non-extension of assistance to this and other sciences by the State is much to be deplored. The Government of a country like ours, whose agricultural, manufacturing, and commercial prosperity is so nearly linked with the advancement of science, extends no helping hand to its votaries, contented with reaping second-hand the benefits of the more liberal policy of other nations! We have great hopes that some good will result from the establishment of the new College of Chemistry;—the principle of Association coming into vicarious operation in lieu of the function which, in a well-regulated society, should be performed by the State.

### DR. HOCKEN ON INFLAMMATORY AFFECTIONS OF THE RETINA.

Dr. Hocken is certainly one of those who have most fully availed themselves of the facilities which medical journalism affords for rushing into print, commented upon by Dr. Cowan. His fertile pen having furnished weeklies, monthlies, bi-monthlies, and quarterlies, with their full share of articles, is now, it seems, enlisted in the service of the annuals; and the present paper, like all his writings, bearing the impress of a talented and observing mind, would have, like most of them, benefited by compression, or perhaps withholding for a season. In fact, we wish our young practitioners would write less and their seniors more; for, while the former are too apt, on the strength of their first few cases, to assume the *ton de professeur*, laying down the law rather too peremptorily for their standing; the latter, far too frequently refrain from imparting to, or leaving for, their brethren the accumulated and invaluable results of years of observation and experience. However, it is time to proceed with our notice of Dr. Hocken's Essay.

Retinitis, or inflammation of the eye, commencing in the Retina, is a rare disease, the membrane probably however frequently becoming affected in the course of the other inflammatory affections of the organ. Dr. Hocken had the opportunity of observing the disease in 20 patients of the 3926 admitted into the West of England Eye Infirmary in the years 1837—41. Of these it occurred six times in the acute and nine times in the chronic form—the form not being specified in the five other cases. Both eyes are seldom affected, at least in the acute form; and it is rare for the inflammation to continue long confined to the retina.

*Acute Retinitis.*—Intense pain and great tenderness of the globe, photophobia, zonular redness of the sclerotica, stopping short of the cornea, impairment of vision, and a greater or less degree of sympathetic fever, denote the *active* form of the disease. Sensations of vivid flashes of fire before the eyes are also observed. The pupil, at first much contracted, afterwards, when the retina exchanges intolerance of, for insensibility to, light, becomes much dilated, presenting a more or less green opacity, according to the nature of the changes operated upon the deep-seated structures. The inflammation may extend to the other textures of the eye, and produce its various consequences. The progress of this form of the disease is very rapid, the eye being soon lost, unless appropriate treatment is resorted to before the disorganizing process is completed. Such cases are however very rare, and the author agrees with Dr. Jacob, that the disease is often mild and insidious, defective vision, rather than symptoms of inflammation, denoting its presence.

" *Passive Form of Acute Retinitis.*—When retinitis occurs in elderly persons, or where the nervous system has been much depressed by mental anxiety and protracted distress; also, where the whole constitution has been much deranged by previous disease; and, lastly, where the original make of the body is feeble; the local phenomena are apt to display much and disorganizing action, without power, and other constitutional symptoms are of a low type. These are very unfavourable cases, having in themselves a greater tendency to an unfavourable termination, and being less under the influence of medicine. In these cases we meet usually with much anxiety and irritability of mind, much languor and depression; the pulse is soft and feeble, sometimes jerking, but readily compressible; the skin dry, but without much heat; and the countenance indicative of depression and anxiety rather than of excitement and fever. The difference between Mr. Tyrrel's experience and my own is, that whilst I should say, from the cases I have seen, that the passive forms of retinitis were the exceptions only, Mr. Tyrrel considers them the ordinary, and more active types, the exceptions to a general rule."

Women seem much more liable to retinitis than men, and most of the subjects of it have become predisposed to disease from their occupations or mode of life. The exciting cause may be sudden exposure to vivid light, long exposure to less intense light, the minute examination of illuminated objects, contusions or wounds of the eye; and "indeed, Dr. Jacob supposes the causes of this affection to be as numerous as those of general inflammation of the eyeball; and, if we allow that retinitis complicates most of the inflammatory affections of the globe, this is doubtless true." In respect to the *diagnosis,* the author observes, he has never seen cases in which the severity of the symptoms could have been mistaken for phrenitis, as mentioned by Mackenzie; but amaurosis proceeding from congestion of the brain might be mistaken for retinitis. In it, however, both eyes are affected, there is not intense pain of the globe or intolerance of light. The vascularity of the eyes is not limited and zonular, while the flashes of light, when they occur, are much less intense. The *prognosis* is very unfavourable, unless the disease be seen in good time, and, even when the symptoms are relieved, relapse is very common.

*Treatment.*—In sthenic forms and plethoric individuals free *bleeding* is required, but must not be indiscriminately resorted to, being inadequate alone to prevent disorganization. "Dr. Jacob says truly, when he observes that daily experience proves how unavailing mere depletion is found in iritis, or general internal inflammation, and even how unsuited to particular cases, however intense the symptoms." In the great majority of cases, local bleeding will suffice, and even this, in the depressed and feeble patient, must be but cautiously resorted to. *Mercury* must be thrown into the system as rapidly, after the relief of vascular tension, as possible, not, however, carrying it to salivation. In slow and protracted cases, *turpentine* may sometimes be advantageously substituted. " In persons far advanced in life, in scrofulous subjects, especially where there is much predisposition to phthisis, and in debilitated persons, this oil is certainly a less hazardous medicine than mercury." The author recommends the formula employed by Mr. Carmichael in iritis. ℞. Ol. Tereb. purif. ℥j. ad ℥iss.; Vitell Ovi; tere et adde Emuls. Amygd. (made with a double portion of confection) ℥iv.; Syr. Aur. ℥ij.; Spt. Lav. Co. ℥iv.; Ol. Cinn. gtt. iv. M. S. coch. ij. ter. Linseed tea or camphor julep to be drank to obviate strangury. Counter-irritation of some kind may be employed behind the ears or at the nape, after the febrile action has become somewhat subdued.

*Chronic Retinitis.*—This may be active or passive in its symptoms, occupying one or both eyes. The sclerotic zone is usually found to be slight, the pupil irregular and discolored, and vision much impaired. The patient is much distressed by sensations of sparks and flashes of various colours, &c. Dull aching pain of the globe, especially at night, is also complained of; it is tender to the touch, being generally firm and swollen, but at other times soft and flaccid. The contraction of the pupil is very remarkable at first, but it becomes dilated and motionless when sensibility of the retina is lost. The progress of the disease is slow, but its ultimate effect is the destruction of vision, having also a tendency to spread to the opposite eye. There is more or less derangement of the system at large, especially of the digestive organs; and in the passive cases there is debility, quick pulse, and hectoid fever. The exciting causes, in most cases, have been the over-strained employment of the eye upon brilliantly illuminated and minute objects. It sometimes follows the acute disease, or may be dependent upon chronic derangement of the health, and the author relates a case in proof of its being sometimes a rheumatic affection. Local bleeding is generally, but not always, advisable, and afterwards mercury or turpentine may be employed according to the activity of the disease and powers of the patient. The mercury, however, must be given so as to produce only the mildest constitutional effects, and therefore much more gradually than in the acute form. The subsequent maintenance of counter-irritation is of importance.

" There is a still more chronic form of retinitis, which I will but briefly sketch. Patients occasionally present themselves, in whom we trace the symptoms of a very chronic form of retinal inflammation; more, in fact, those of retinal hyperæmia than inflammation, being very slow in their progress, and ultimately inducing changes different from the usual effects of chronic retinitis. This form of disease is scarcely, if ever, spoken of as retinitis, but placed under the indefinite term of 'amaurosis,' which, like many other terms, serves as a convenient cloak for ignorance or sloth. It is not by any means a very common disease *per se*, but comparatively so, in common with active or passive cerebral congestion. In this variety of retinitis the eye has a dull, congested, appearance, the conjunctival and sclerotic vessels are enlarged, vision fails gradually, attended with luminous appearances before the eyes, muscæ volitantes, a distorted, broken image in the perception of objects, &c. whilst a gradual change of colour is effected in the pupil, which becomes misty, and presents a turbid, somewhat greenish hue. Here there is no stage of intolerance of light, but from the beginning diminished sensibility of the retina, and desire for bright light. In proportion as the blindness increases so the pupil dilates, and moves with a sluggish and very imperfect motion. In general hyperæmia of the visual textures, both eyes are simultaneously affected; in this chronic form of retinitis, usually but one; but, after the continuance of the disease for some time, its fellow frequently suffers from a similar disease of the same character and parts. All the usually described symptoms of amaurosis may be present in this disease, or any or most of them may be absent. Although very chronic and intractable, frequently terminating in incurable disorganization, yet, when actively and timely treated by an antiphlogistic and mercurial plan, many are brought back to the precincts of health. Mercury, unless very early and steadily used, is less effective than in the more acute cases, since the very actions which disorganize, render these conditions permanent during the continuance of the disease itself."

### An Essay, Literary and Practical, on Inversio Uteri. By *John Green Crosse.*

Here we have a production from the pen of one of the seniors of the profession, and we shall be probably thought captious in stating it as scarcely such a one as we could have expected or desired from such a source. Practical observations upon this, or upon any subject, from so able a practitioner as Mr. Crosse, will always command due attention; but we are inclined to believe the "laborious research for several months past" in accumulating materials for the literary portion of the Essay might have been spared; for, notwithstanding a most unwieldy display of lengthy footnotes, we do not find any very novel view of a subject already well understood displayed, or authorities with which the profession was not already well acquainted with cited. It is true we have here only the commencement of the Essay, the present sixty pages or so, being occupied in the description of the varieties of the disease and their symptoms.

Treating first of inversion occurring after parturition, Mr. Crosse divides it into its various degrees, depression, introversion, perversion, and total inversion. Simple *depression* of a portion of the fundus may give rise to serious or even fatal hæmorrhage, but is not of long persistence, passing, if not relieved, into the next stage, *introversion*, in which the fundus and a portion of the body of the organ is "received into the remainder of the body and cervix, the convexity of the fundus being palpable at the os tincæ." When more or less of the inverted portion passes through the os tincæ, *perversion* is said to exist; and the whole fundus and body may do this, leaving only the cervix in situ.

"But if the labia resist sufficiently the descent of the fundus, and part of the body remains still uninverted, may not the process be carried to its completion by ascent of the cervix? No author has hinted at this view of the subject, and yet its correctness must be admitted, in order to explain the well-established fact, that, where the inverted fundus and body are still in the vagina, the cervix is felt high above the pubes, even near to the navel, sometimes taking the situation the fundus would normally occupy, the vagina being proportionally stretched and carried upwards;—changes which can only be explained by supposing that, at a certain stage, the inversion ceased to progress by descent of the fundus, and was continued and completed by ascent of the cervix."

The uterus may take more or less time in the passage through these various stages of partial inversion. It may accomplish it instantly under the influence of expulsive efforts or traction at the umbilicus. On the other hand, days or weeks may be occupied in the conversion of a depression or introversion into a perversion; and the cases recorded of inversion occurring several days after labour are probably to be explained by a slight degree of it having been at first overlooked. In *total inversion* the cervix as well as the other parts of the uterus is inverted, and although the possibility of this was denied by Baudelocque and others, a host of modern writers have testified to its occurrence, and among others the author himself, in a case narrated by him in the Provincial Journal of last year. Prolapse has been frequently confounded with the various degrees of inversion. It may be added to partial, and is still more commonly so, to total inversion; but this may not take place until days, or even weeks after delivery. It always much aggravates the suffering and danger of the patient.

There is no evidence of inversion ever having taken place from the cervix or body towards the fundus, at least in the human subject; but

"Several of the domesticated quadrupeds are liable to uterine inversion, with prolapse; and I have reason to believe that the progress of the malady in them is often, if not uniformly, from the vaginal termination of the uterus towards the fundus, the reverse of what happens with woman. Many authors who have attempted to describe the progress of inversion, have compared it to 'turning of the finger of a glove inside out' on removing it from the hand; the one is just the reverse of the other, in the human frame, as to the successive changes, although the effect may be the same when, in both instances, the inversion is rendered complete. The comparison would be correct only on the condition that uterine inversion progressed from the cervix to the fundus."

The lining membrane of the inverted uterus, unless inflamed or otherwise morbidly affected, possesses little sensibility; and in the case, occurring to the author, where total inversion had existed for several days, he was enabled to scratch, prick, and apply ice to the membrane without exciting sensation. If the patient survive the first shock and the uterus be not reduced, the hæmorrhage and constitutional symptoms may, as well as the fetid discharge, subside, the organ also gradually diminishing in size, until it does not exceed that of the unimpregnated uterus. It may now be considered a case of *chronic inversion*, perversion being the form usually found to be present. The tumour is of a pale-red or florid colour, covered with mucus, and easily bleeding. It is dense and firm, but larger and softer during menstruation.

"In all instances where the cervix only remains uninverted, the tumour is smaller at its highest part next the cervix, and increases in size as you approach its centre; whatever some writers have stated to the contrary, I find no exception to the correctness of the remark, that the circumference of the tumour is greatest midway between its two extremities, and that a gradual diminution is observable as you trace the tumour upwards towards the encircling cervix."

This observation, however, only applies as long as the organ continues in a quiescent state, the symptoms induced being mild and not inconsistent with the continuance of active life. When, from hæmorrhages, or other causes, the health becomes injured, or the uterus becomes inflamed or ulcerated, or suffers constriction from the cervix, its appearance and shape become proportionally altered; or, indeed, the organ may never have diminished to its natural size by reason of the influence of some of these causes. In the majority of instances prolapse takes place sooner or later.

The presence of *polypus*, is, after pregnancy, the most frequent cause of inversion, and Velpeau has related a case in which a polypus the size of a finger attached to the fundus produced this effect. The author also relates a case of fungoid disease, by which inversion was caused. So, too, the removal of polypi or other large uterine tumours may be attended with inversion. A case is briefly detailed which occurred to Mr. Johnson, one of the author's colleagues at the Norfolk Hospital. An enormous tumour, weighing 32 ozs. was removed by ennucleating a portion from day to day.

"When the last part of the tumor passed the external labia, the operator, who alone could be cognizant of this circumstance, felt the inverted portion of the uterus in the vagina, and judged its thickness to be not more than $\frac{1}{8}$ inch. It was pushed up, so as to be in some degree replaced in the pelvis, and above the os; and although so thin when expanded, the uterine wall must have contracted, for Mr. Johnson assures me that, on examination after the patient's perfect recovery, there was not the slightest projection or irregularity observable, the uterus occupying the same space, and presenting the same shape, as in health."

A case of inversion of the uterus, produced by the expulsion of hydatids, and the subsequent successful removal of the organ by ligature, related by Dr. Thatcher, of Edinburgh, in his lectures, is quoted. But a small portion of the author's task is finished with this paper, and for its completion he says, " until each succeeding section is actually written and in type, I shall assiduously employ myself in collecting further information, and thankfully receive it, through any of the numerous channels which have been so promptly and so liberally opened to me, not only in the United Kingdom, but in various parts of Europe, and in far more distant countries."

The portion that is now before us is ably written, and several well-executed lithographs represent various of the preparations referred to; but Mr. Crosse seems to us to have treated the subject as if he believed the nature of the affection to be far less familiarly known to the profession than is really the case.

For a description of Mr. Giles's case of Congenital Malformation of the Male Urinary Organs we must refer to the volume itself.

